# Supplementary figures and images for: Phosphoproteomic analysis of lettuce (Lactuca sativa L.) reveals starch and sucrose metabolism functions during bolting induced by high temperature
Source: PLoS One. 2020 Dec 29;15(12):e0244198. doi: 10.1371/journal.pone.0244198 (PMC7771692; doi:10.1371/journal.pone.0244198)

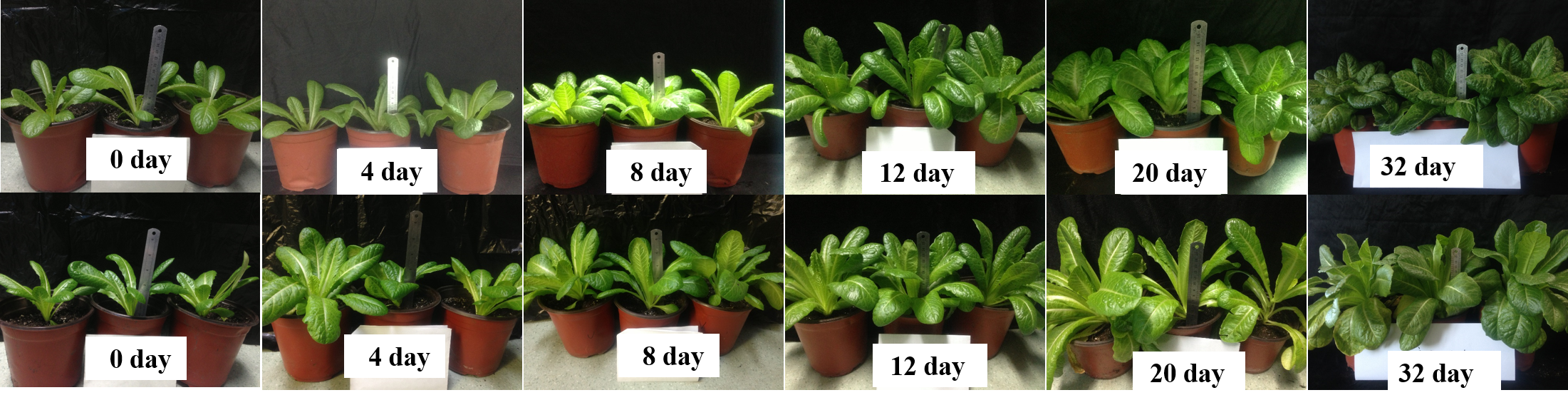

Supplement: S1 Fig — (TIF) [file pone.0244198.s003.tif]
